# COVID-19 vaccination rates in hospitalized mentally ill patients compared to the general population in Germany: Results from the COVID Ψ Vac study

**DOI:** 10.1192/j.eurpsy.2022.33

**Published:** 2022-06-28

**Authors:** Hauke F. Wiegand, Birgit Maicher, Mike Rueb, Paula Wessels, Bianca Besteher, Sabine Hellwig, Andrea Pfennig, Henrik Rohner, Stefan Unterecker, Lars P. Hölzel, Alexandra Philipsen, Katharina Domschke, Peter Falkai, Klaus Lieb, Kristina Adorjan

**Affiliations:** 1 Department of Psychiatry and Psychotherapy, University Medical Center of the Johannes Gutenberg-University, Mainz, Germany; 2 Department of Psychiatry and Psychotherapy, Carl Gustav Carus University Hospital, Medical Faculty, Technische Universität, Dresden, Germany; 3 Department of Psychiatry and Psychotherapy, University Hospital, LMU Munich, Munich, Germany; 4 Institute for Medical Information Processing, Biometry and Epidemiology, LMU Munich, Munich, Germany; 5 Pettenkofer School of Public Health, LMU Munich, Germany; 6 Department of Psychiatry, Psychosomatics and Psychotherapy, University Hospital of Würzburg, Würzburg, Germany; 7 Department of Psychiatry and Psychotherapy, Jena University Hospital, Jena, Germany; 8 Department of Psychiatry and Psychotherapy, Medical Center—University of Freiburg, Faculty of Medicine, University of Freiburg, Freiburg, Germany; 9 Department of Psychiatry and Psychotherapy, University Hospital of Bonn, Bonn, Germany; 10 Oberberg Parkklinik Wiesbaden Schlangenbad, Schlangenbad, Germany

**Keywords:** vaccination, vaccination rates, mental illness, inpatient, COVID-19

## Abstract

**Background:**

Mental illness is known to come along with a large mortality gap compared to thegeneral population and it is a risk for COVID-19 related morbidity andmortality. Achieving high vaccination rates in people with mental illness is therefore important. Reports are conflicting on whether vaccination rates comparable to those of the general population can be achieved and which variables represent risk factors for nonvaccination in people with mental illness.

**Methods:**

The COVID Ψ Vac study collected routine data on vaccination status, diagnostic groups, sociodemographics, and setting characteristics from in- and day-clinic patients of 10 psychiatric hospitals in Germany in August 2021. Logistic regression modeling was used to determine risk factors for nonvaccination.

**Results:**

Complete vaccination rates were 59% (*n* = 776) for the hospitalized patients with mental illness versus 64% for the regionally and age-matched general population. Partial vaccination rates were 68% (*n* = 893) for the hospitalised patients with mental illness versus 67% for the respective general population and six percentage (*n* = 74) of this hospitalized population were vaccinated during the hospital stay. Rates showed a large variation between hospital sites. An ICD-10 group F1, F2, or F4 main diagnosis, younger age, and coercive accommodation were further risk factors for nonvaccination in the model.

**Conclusions:**

Vaccination rates were lower in hospitalized people with mental illness than in the general population. By targeting at-risk groups with low-threshold vaccination programs in all health institutions they get in contact with, vaccination rates comparable to those in the general population can be achieved.

## Introduction

Mental illness is conceived a risk for COVID-19 related morbidity and mortality. Studies agree that patients with psychotic and to a lower degree mood disorders seem to be at risk for COVID-19 associated morbidity and mortality [1, 2] and some studies show a higher risk for addiction disorders [3]. This risk status seems to be mostly related to common somatic comorbidities like metabolic syndrome, cardiovascular, and respiratory disorders associated with mental disorders due to reduced self-care, medication side effects, more precarious social and living conditions, and insufficient somatic medicine treatment [1–4]. By these risk factors in combination with pandemic-associated reduced health-care services [5, 6] the COVID-19 pandemic has the potential to further broaden the appallingly large mortality gap for severe mental disorders [7, 8]. Therefore, high vaccination rates among the risk groups with mental illness are of high public health priority. In the initial COVID-19 vaccination campaign in 2021, in some countries like Denmark, the Netherlands, the UK, and Germany populations with severe mental illness were prioritized for vaccination [9] but it remained unclear to what degree at-risk groups with mental illness were reached by vaccination campaigns in every-day routine health-care practice. Studies on vaccination rates of populations with mental illness in comparison to the general population showed heterogenous results: In some studies vaccination rates were comparable to or better than the general population [10–13] but in others people with mental illness showed much lower vaccination rates [14–17]. A common factor for vaccination rates similar to the general population seemed to be vaccination offerings by mental health institutions [10–12]. This corresponds to studies on reasons for nonvaccination that did not show a general vaccine hesitancy among people with mental illness but were organizational access issues appeared to be the most relevant factors [15, 17–19]. Some studies on risk groups for nonvaccination identified a younger age [12, 17] and a schizophrenia diagnosis [13, 15, 16] associated with nonvaccination while others found no such differences [11, 20].

Therefore, the first aim of the COVID Ψ Vac study was to determine vaccination rates among hospitalized people with mental illness in routine health care in comparison to the age-equivalent general population in Germany at a time period in August 2021, when access to COVID-19 vaccines was widely available. The second aim was to identify risk populations for unvaccinated status by available routine data indicators in order to identify target groups for vaccination programs among populations with mental illness.

## Methods

### Study design and participants

Ten psychiatric hospitals and departments in five regions of Germany took part in the study. Seven departments were part of university medical centers. They all had a regional care mandate (“Versorgungsauftrag”), what means that they were responsible for the inpatient psychiatric emergency care of a defined region and that they cannot selectively choose but have to admit all patients with an indication from this region. For achieving a rapid data collection in the evolving pandemic the choice of hospitals was a convenience sample based on participation in the NUM egePan COVID-19 research network that funded the study and willingness to participate in the study. Six of the 10 hospitals offered in-hospital COVID-19 vaccinations during inpatient mental health treatment, in those facilities all patients got weekly vaccination offerings. Between August 4, 2021 and August 19, 2021, a time period were COVID-19 vaccines were widely available for the adult population, the participating institutions selected a reference date and collected the bellow mentioned routine data of all inpatient and day-clinic patients in treatment at this day. For assessing representativity, the sample’s main diagnoses and age groups were compared to the latest version of the publicly available Germany-wide hospital statistic (“Krankenhausstatistik”) from 2019 of the German Federal Statistical Office (“Statistisches Bundesamt”) [21]. For comparing vaccination rates between the population hospitalized for mental illness and the general population, the regionally and age-matched general population vaccination rates were calculated using public data from the German Robert Koch Institute (RKI) from August 12, 2021 as a reference [22]. The RKI is Germany’s public health institute. Its vaccination statistic (“Impfquotenmonitoring”) is the most reliable publicly available source based on a mandatory electronic reporting of all COVID-19 vaccinations. However, the RKI itself assumes a modest but tolerable underreporting based on comparisons with vaccine delivery and accounting data.

### Routine data indicators and outcomes

The following routine data variables were collected for all inpatient and day clinic services, they were chosen based on availability: Age categorical (18–39, 40–60, 60+); gender (female, male, and diverse); ICD-10 main diagnosis groups (F0: organic mental disorders, F1: addictive disorders, F2: psychoses, F3: affective disorders, F4: anxiety, obsessive-compulsive, stress-associated, dissociative, and somatoform disorders, and F6: personality disorders); presence of somatic comorbidities (adapted from the RKI’s list of risk conditions for severe SARS-CoV-2 outcomes), inpatient or day clinic setting; hospital stay >3 months; admission mode acute or elective, legal status “voluntary” or “coercive accommodation”; residential status “independent,” “assisted,” or “homeless”; and COVID-19 vaccination status “unvaccinated,” “partially vaccinated,” “fully vaccinated,” or “recovered.” The RKI list of risk conditions for severe SARS-CoV-2 outcomes comprised the following conditions: obesity with BMI >30, diabetes mellitus, disorders of the cardiovascular system (arterial hypertension, coronary heart disease, etc.) chronic lung diseases (COPD, etc.), neoplasms, chronic kidney and liver diseases, weakened immune system (by disease or medication).

### Statistical analysis

To determine risk factors of the outcome “vaccination status unvaccinated,” a logistic regression with a multilevel random intercept model with “hospital site” as random effect was chosen because of categorical variables and high variation between hospital sites characteristics. To estimate the variance explained by the random effect “hospital site,” the Intraclass Correlation Coefficient was calculated. All other variables were then included in a model as fixed effects. In each case, the reference category used was the one with the highest vaccination rate (e.g., F3 for ICD-10 categories, see Table 1). To examine the goodness of fit of the model, Nakagawa’s marginal and conditional *R*^2^ were used [23]. All calculations were performed in Rstudio 1.4.1717 using the “base,” “datasets,” and “lme4” packages.

**Table 1.** Demographic and Clinical Characteristics of the Study Sample

	Total		Recovered from SARS-CoV-2 infection		Partially vaccinated		Fully vaccinated		Unvaccinated	
	*n*	%	*n*	%	*n*	%	*n*	%	*n*	%
Total	1,322	100	21	2	117	9	776	59	408	31
Gender										
F	711	54	13	2	62	9	415	58	221	31
M	609	46	8	1	55	9	359	59	187	31
Age group										
18–39	538	41	8	1	56	10	275	51	199	37
40–60	437	33	7	2	39	9	274	63	117	27
60+	347	26	6	2	22	6	227	65	92	27
Residential status										
Independent	1,148	87	20	2	110	10	683	59	335	29
Assisted	133	10	0	0	2	2	80	60	51	38
Homeless	37	3	X	X	5	14	20	54	11	30
Main diagnosis										
F0	95	7			3	3	58	61	33	35
F1	207	16	4	2	16	8	107	52	80	39
F2	276	21	2	1	34	12	126	46	114	41
F3	593	45	10	2	56	9	397	67	130	22
F4	132	10	3	2	8	6	74	56	47	36
F6	17	1	X	X	0	0	12	71	4	24
Somatic comorbidities										
No	712	54	13	2	78	11	369	52	252	35
Yes	610	46	8	1	39	6	407	67	156	26
Setting										
Inpatient	1,115	84	20	2	98	9	639	57	358	32
Day clinic	207	16	1	0	19	9	137	66	50	24
Length of stay >3 month										
No	1,229	93	19	2	112	9	723	59	375	31
Yes	93	7	2	2	5	5	53	57	33	35
Admission mode										
Elective	722	55	11	2	69	10	458	63	184	25
Acute	600	45	11	2	48	8	318	53	224	37
Legal status										
Voluntary	1,205	91	19	2	111	9	733	61	342	28
Coercive accommodation	117	9	2	2	6	5	43	37	66	56

### Ethics, data protection, and funding

The COVID Ψ Vac study was part of the BMBF-funded egePan collaborative project within the German National Network University Medicine (NUM), a network for COVID-19-related research. Positive votes from the regional ethics committees responsible for the participating institutions as well as the data protection department of the University Medicine Mainz were available. *N* = 88 patients had to be excluded for data protection reasons because they would have been individually identifiable based on the routine data variables.

## Results

### Population characteristics and sample representativity

Routine data from *n* = 1,320 patients was included in the study, 54% (*n* = 711) were female, 41% (*n* = 538) between 18 and 39 years, 33% (*n* = 437) between 40–60 and 26% (*n* = 347) above 60 years of age. Eighty-seven percentage (*n* = 1,148) were living independently, 10% (*n* = 133) in assisted living facilities and 3% (*n* = 37) were homeless. Seven percentage (*n* = 95) had an ICD-10 F0 main diagnosis, 16% (*n* = 207) an ICD-10 F1 main diagnosis, 21% (*n* = 276) an F2 main diagnosis, 45% (*n* = 593) an F3 main diagnosis, 10% (*n* = 132) an F4 main diagnosis and 1% (*n* = 17) an F6 main diagnosis. Forty-six percentage (*n* = 610) had a known somatic comorbidity from the RKIs list of risk conditions for severe SARS-CoV-2 outcomes. Eighty-four percentage (*n* = 1,115) were in inpatient treatment, 16% (*n* = 207) in day-clinic treatment; 9% (*n* = 117) were coercively accommodated. Fifty-five percentage (*n* = 722) were admitted electively to inpatient or day-clinic treatment, 45% (*n* = 600) had an acute admission. Seven percentage (*n* = 93) had been in inpatient or day-clinic treatment for more than 3 months (Table 1).

Table 2 shows the sample’s gender, age groups and ICD-10 main diagnoses compared to the Federal Statistics Office’s statistic on all German mental health inpatient facilities in 2019. The two samples were largely comparable, except for the COVID Ψ Vac sample having slightly more patients in the 18–39 years group (41 vs. 34%) and slightly less patients in the 40–59 years group (33 vs. 40%).

**Table 2.** Comparison of Study Sample and Hospital Statistic 2019 Sample

	COVID Ψ Vac sample (in %)	Hospital statistic 2019 of the federal statistics office sample^a^ (in %)
Gender		
F	54	53
M	46	47
Age group		
18–39	41	34
40–60	33	40
60+	26	26
Main diagnosis		
F0	7	8
F1	16	16
F2	21	19
F3	45	42
F4	10	10
F6	1	5

a The hospital diagnosis statistics is an annual census of patients who were discharged from inpatient treatment in a hospital in Germany in the reporting year. It contains data of all hospitalized patients in Germany. The table shows the results filtered for the ICD-10 groups selected in the COVID Ψ Vac study.

### Vaccination rates

The overall complete vaccination rate among hospitalized patients with mental illness was 59% (*n* = 776) with a large range between hospital sites of 32–71%. Three percentage (*n* = 41) were vaccinated during the hospital stay. Two percentage (*n* = 21) were recovered from a SARS-CoV-2 infection within the last 6 month and thus not eligible for vaccination. The regionally and age-matched general population complete vaccination rate was 64% [22]. Sixty-eight percentage (*n* = 893) of the hospitalized SMI patients were vaccinated at least once. Six percentage (*n* = 74) with partial vaccination were vaccinated during the hospital stay. In the regionally and age-matched general population 67% were vaccinated at least once.

Comparing main diagnosis groups, complete vaccination rates were highest with 71 and 67% for patients with an F6 (but very low n) and an F3 main diagnosis respectively and with 46% lowest for patients with an F2 main diagnosis.

In the age group 60+ vaccination rates were the highest with 65% (*n* = 227) completely vaccinated and 71% (*n* = 249) at least partially vaccinated. In the 40–60 years age group 63% (*n* = 274) were completely vaccinated, 71% (*n* = 313) at least partially vaccinated. In the 18–39 age group 51% (*n* = 275) were completely vaccinated, 61% (*n* = 331) at least partially vaccinated (Table 1).

### Risk factors for being unvaccinated

To determine risk factors of the outcome “vaccination status unvaccinated,” a logistic regression with a multilevel random intercept model with “hospital site” as random effect was chosen. Patients with the gender “diverse,” “ICD-10 group F6,” and residence status “homeless” were excluded from regression because of too small group sizes. All other variables were then included in a model as fixed effects. In each case, the reference category used was the one with the highest vaccination rate. The ICD-10 categories F1, F2, and F4, age category 18–39, absence of somatic comorbidities, and legal status “coercive accommodation” showed significant effects (Table 3). Nakagawa’s marginal *R*^2^ was 0.12, the conditional *R*^2^ 0.22. The Intraclass Correlation Coefficient was calculated and 11.4% of the variance was explained by the random effect “hospital site.”

**Table 3.** Logistic Regression Model for “Vaccination Status Unvaccinated”

Random effect		
Variable	Variance	SD
Hospital site	0.424	0.6511

Fixed effects		
Variable	Odds-ratio	*p*-value
Intercept	0.12 (0.06–0.24)	<0.001***
ICD-10 F0	1.22 (0.65–2.21)	0.53
ICD-10 F1	2.43 (1.62–3.64)	<0.001***
ICD-10 F2	1.65 (1.14–2.39)	<0.01**
ICD-10 F4	1.81 (1.15–2.84)	<0.01**
Age 18–39	1.88 (1.22–2.93)	<0.01**
Age 40–60	1.12 (0.74–1.71)	0.60
Sex male	0.96 (0.73–1.26)	0.76
Somatic comorbidities	1.55 (1.15–2.10)	<0.01**
Acute admission	1.31 (0.93–1.83)	0.12
Coercive accommodation	3.46 (2.05–5.92)	<0.001***
Inpatient setting	1.37 (0.90–2.10)	0.14
Assisted living	0.99 (0.63–1.52)	0.95
Length of stay >3 month	1.16 (0.68–1.96)	0.58

Because of the inevitable collinearity between the variables “age group” and “ICD-10 diagnostic group” we calculated the same model again just for the age group 40–60, in which all ICD-10 diagnostic groups were represented in significant numbers. For the ICD-10 categories F1 (odds ratio OR 2.92 [1.44–5.90], *p* = 0.003), F2 (OR 2.15 [1.07–4.32], *p* = 0.03), and F4 (OR 10.55 [3.97–28.02], *p* < 0.001) absence of somatic comorbidities (OR 1.73 [1.03–2.90], *p* = 0.04) and legal status “coercive accommodation” (OR 7.50 [2.43–23.11], *p* < 0.001) effects remained significant. Additionally, residential status “assisted” (OR 0.28 [0.10–0.83], *p* < 0.001) showed a significant effect.

## Discussion

The results show mediocre vaccination rates of 59% in the fairly representative sample of hospitalized patients with mental illness and of 64% in the regionally and age-matched general population in Germany. Three observations are especially of interest when discussing vaccination rates and strategies for risk groups among mentally ill people.

First, the results show a lower full-vaccination rate for the hospitalized mentally ill compared to the general population (59 vs. 64%) but a higher partial vaccination rate (68 vs. 67%). This effect was largely due to in-hospital vaccination programs in some of the participating institutions, where patients hospitalized for mental illness got weekly vaccination offerings. Thereby, these routine care results confirm studies, which attributed lower vaccination rates in those with mental illness mainly to access barriers and not to a generally higher vaccination unwillingness [10–12, 17–19, 24]. They highlight the need to offer people with mental disorders repeatedly and actively COVID-19 vaccinations at all those health care providers, where they are in trusting and stigmatization-free contact with the health care system, thus psychiatric hospitals, outpatient clinics, and office-based psychiatrists and not only at centralized vaccination facilities or somatic medicine providers.

Second, the study identified risk factors for nonvaccination, namely a younger age, a principal diagnosis of addictive disorder, psychosis or F4 group disorder (anxiety, obsessive-compulsive, stress-related, dissociative, and somatoform disorders) and coercive accommodation status. Psychoses have been identified as a risk factor for nonvaccination in other studies [15, 16] and addiction has been associated with poorer COVID-19 outcomes [25]. The results of lower vaccination rates despite a higher risk correspond to findings of a poorer quality of physical health care in general in exactly these severely mentally ill populations despite a high burden of physical disorders and an alarmingly huge mortality gap compared to the general population [26]. Therefore, for preventing a further widening of the preexisting mortality gap in severely mentally ill people by the COVID-19 pandemic, mental health care and somatic medicine, including vaccination offerings, need to be better integrated in the future and mental health care facilities should routinely offer basic somatic medical care.

Third, despite Germany being a high-income country with universal health insurance coverage and during the data collection period in August 2021 widely available COVID-19 vaccines, vaccination rates were (and are still) quite low in comparison to, for example, in France or Denmark. Furthermore, a remarkably high regional variation was observable both in the population of hospitalized people with mental illness and in the general population. This fits other studies results that highlight the importance of regionally variable attitudes toward vaccinations.

This study has several limitations: The convenience sample of hospital sites with a large share of university hospitals was a compromise for gaining a large enough sample rapidly in the light of the rapidly evolving pandemic in a country with unfortunately no routinely available access to routine data for research purposes and strict data protection laws. The use of quite coarse routine data variables collected for other purposes can give information about vaccination rates and diagnostic groups but not about subjective factors for nonvaccination. Therefore, further qualitative research on these factors is very important for identifying, understanding and addressing these factors in order to further boost vaccination rates. A further limitation is the exclusion of 6% (*n* = 88) of the hospitals’ patients, a compromise that had to be made for balancing needs for on the one hand data protection and on the other hand rapid study implementation. This exclusion might have led to bias or obscuration of smaller risk groups. However, regarding gender, age and ICD-10 diagnostic groups the studies sample can be regarded as representative for hospitalized patients with mental illness in Germany and the findings concerning vaccination rates, risk groups and the effect of in-hospital vaccination strategies should be fairly valid.

Currently, all over Europe COVID-19 infection protection and prevention measures are lifted. However, the COVID-19 pandemic is not over, incidences are still high and infections can still be deadly in unvaccinated risk groups. Therefore, especially in countries with a low general population vaccination rate like Germany, it must remain a continuous top public health priority to systematically protect vulnerable and stigmatized risk groups like people with severe mental illness. As people with mental illness often engage more readily with mental health than with somatic medicine providers, psychiatric hospitals, outpatient clinics, and office-based psychiatrists should be enabled systematically to provide vaccinations in order to prevent the mortality and morbidity gap of mentally ill populations to further widen by the pandemic.

## Data Availability

The data that support the findings of this study are available on request from the corresponding author (H.F.W.) within the limits of data protection. The data are not publicly available due to data protection requirements regarding individual patient routine data.
